# Improving the utility of the Brunnstrom recovery stages in patients with stroke: Validation and quantification

**DOI:** 10.1097/MD.0000000000004508

**Published:** 2016-08-07

**Authors:** Chien-Yu Huang, Gong-Hong Lin, Yi-Jing Huang, Chen-Yi Song, Ya-Chen Lee, Mon-Jane How, Yi-Miau Chen, I-Ping Hsueh, Mei-Hsiang Chen, Ching-Lin Hsieh

**Affiliations:** a Department of Occupational Therapy, I-Shou University, Kaohsiung, Taiwan; b School of Occupational Therapy, College of Medicine, National Taiwan University; c Department of Health Promotion and Gerontological Care, Taipei College of Maritime Technology, Taipei; d Department of Occupational Therapy, College of Medical and Health Science, Asia University, Taichung; e Department of Physical Medicine and Rehabilitation, National Taiwan University Hospital, Taipei; f School of Occupational Therapy, Chung Shan Medical University; g Occupational Therapy Room, Chung Shan Medical University Hospital, Taichung, Taiwan.

**Keywords:** brunnstrom recovery stages, quantification, stroke, validation

## Abstract

The Brunnstrom recovery stages (the BRS) consists of 2 items assessing the poststroke motor function of the upper extremities and 1 assessing the lower extremities. The 3 items together represent overall motor function. Although the BRS efficiently assesses poststroke motor functions, a lack of rigorous examination of the psychometric properties restricts its utility. We aimed to examine the unidimensionality, Rasch reliability, and responsiveness of the BRS, and transform the raw sum scores of the BRS into Rasch logit scores once the 3 items fitted the assumptions of the Rasch model.

We retrieved medical records of the BRS (N = 1180) from a medical center. We used Rasch analysis to examine the unidimensionality and Rasch reliability of both upper-extremity items and the 3 overall motor items of the BRS. In addition, to compare their responsiveness for patients (n = 41) assessed with the BRS and the Stroke Rehabilitation Assessment of Movement (STREAM) on admission and at discharge, we calculated the effect size (ES) and standardized response mean (SRM).

The upper-extremity items and overall motor items fitted the assumptions of the Rasch model (infit/outfit mean square = 0.57–1.40). The Rasch reliabilities of the upper-extremity items and overall motor items were high (0.91–0.92). The upper-extremity items and overall motor items had adequate responsiveness (ES = 0.35–0.41, SRM = 0.85–0.99), which was comparable to that of the STREAM (ES = 0.43–0.44, SRM = 1.00–1.13).

The results of our study support the unidimensionality, Rasch reliability, and responsiveness of the BRS. Moreover, the BRS can be transformed into an interval-level measure, which would be useful to quantify the extent of poststroke motor function, the changes of motor function, and the differences of motor functions in patients with stroke.

## 1 Introduction

Motor recovery is one of the most important treatment goals for patients with stroke.^[1]^ A valid, reliable, and responsive measure assessing poststroke motor function is essential for appropriate clinical decision-making, treatment planning, and research (e.g., outcome studies). The Brunnstrom recovery stages (BRS) is a short and easily administered measure for assessing motor function.^[2]^ The BRS contains 3 items for the arm (BRS-A), the hand (BRS-H), and the leg (BRS-L), all of which are rated on a 6-level Likert-type scale. These items are usually used individually to describe the motor function (i.e., the arm, the hand, and the leg, respectively) of a patient. The BRS has good item-level psychometric properties.^[3–7]^ Therefore, the BRS may be useful in both clinical and research settings.

However, 3 weaknesses restrict the utility of the BRS. First, the unidimensionality of the BRS has not yet been investigated. It is unknown whether the 3 items of the BRS assess the same construct and whether the scores of the items can be summed to represent overall motor function.^[8]^ Sum scores of the BRS could quickly provide an overall impression of a patient's motor function as an alternative to inspecting the score of each item. Moreover, sum scores could be an outcome indicator because any progress made on each item by a patient could be detected, which is useful for monitoring a patient's overall change over time and determining the effects of intervention. Therefore, validation of the unidimensionality of the BRS is warranted.

Second, it is unknown whether the BRS is as responsive as lengthier measures of motor function, such as the stroke rehabilitation assessment of movement (STREAM), which has moderate to large responsiveness.^[14,15]^ Theoretically, the STREAM would be more responsive than the BRS because the STREAM contains more items for detecting changes in motor function. However, previous studies have shown that the responsiveness of the short-form format of a measure can be comparable to that of the long-form format in a group of patients.^[9,10]^ Since the BRS has high feasibility, and if it is as responsive as the STREAM, the BRS would be appropriate as an outcome measure for a group of patients to decrease the evaluation burden on both patients and users.

Third, the BRS is rated on an ordinal scale rather than on an interval scale. An ordinal scale identifies the order of the values, but the differences between the values remain unknown.^[11]^ An interval scale not only identifies the order of the values but also has equal intervals between any 2 adjacent values.^[11,12]^ In the case of the BRS, scores rated on an interval scale would be helpful in both quantifying the changes in motor function of a patient and comparing the differences in motor function between patients.

Rasch analysis is based on a mathematical model that can estimate person ability (the motor function in our study) and item difficulty (the level of difficulty of each item) simultaneously, and then place the person ability and the item difficulty on the same interval scale.^[13]^ This method has 3 advantages. First, items that fit the assumptions of the Rasch model are unidimensional, which facilitates examination of the unidimensionality of a measure. Second, Rasch analysis helps users transform an ordinal-level measure into an interval measure. Rasch scores, scored on an interval scale, can be used to represent the estimated person ability and item difficulty. Third, Rasch analysis provides Rasch reliability, which is an indicator of measurement error of the BRS scores. Measurement error is useful in determining whether the motor functions measured by the BRS are close to patients’ real motor functions.

Although BRS efficiently assesses motor functions, its utility is restricted because its psychometric properties have not been rigorously examined. This study had 2 purposes. First, we aimed to examine the psychometric properties of the BRS, including the unidimensionality, Rasch reliability, and responsiveness. Second, once the 3 items fitted the assumptions of the Rasch model, we aimed to transform the sum scores of the 3 items (an ordinal scale) into Rasch logit scores (an interval scale). We hypothesized that both the upper-extremity items and the overall motor items of the BRS were unidimensional, the responsiveness of the BRS was comparable with that of the STREAM, and the BRS could be transformed from an ordinal-level measure into an interval-level measure.

## 2 Methods

### 2.1 Participants

We retrospectively retrieved a set of admission and discharge data from 2012 to 2014 from medical records of the occupational therapy (OT) department of a medical center. We selected medical records according to the following inclusion criteria: patients who had diagnoses of stroke, and patients who underwent BRS evaluations at admission. Diagnoses of stroke were based on the *International Classification of Disease, Ninth Revision, Clinical Modification Codes*, including cerebral hemorrhage (431), cerebral infarction (434), or others (430, 432, 433, 436, and 437).

This study was approved by the institutional review board of the medical center.

### 2.2 Measures

The BRS was designed to describe a sequence of extremity motor recovery after stroke based on the synergy pattern of movement that develops during recovery from a flaccid limb to near-normal and normal movement and coordination.^[2]^ The BRS contains 3 items: BRS-A, BRS-H, and BRS-L, which are scored on a 6-level Likert-type scale (level I to VI). Higher levels represent better motor function. Clinicians rate a patient's stage based on the patient's spasticity and movement. It takes less than 10 minutes to complete the evaluation. In our study, levels I to VI were respectively recoded into scores of 0 to 5 for Rasch analysis.

The STREAM was designed to provide a comprehensive, objective, and quantitative evaluation of the motor functioning of patients with stroke.^[14]^ It is a user-friendly measure because the content and format of the STREAM were created in an effort to minimize barriers to routine clinical use. The STREAM contains 30 items divided into 3 subscales: 10 items for voluntary motor ability of the upper-extremity, 10 items for voluntary motor ability of the lower-extremity, and 10 items for basic mobility. The upper-extremity and the lower-extremity subscales were used in our study. A 3-point ordinal scale is used for scoring voluntary movement of the extremities. The STREAM has excellent reliability (intraclass correlation coefficients ≥0.97), good concurrent validity with the Fugl-Meyer motor assessment, satisfactory predictive validity (ρ = 0.69–0.75), and moderate to large responsiveness (effect size (ES) d = 0.47–0.51; standardized response mean (SRM) = 0.83–1.00).^[15]^

### 2.3 Statistical analyses

#### 2.3.1 Descriptive analyses

Descriptive statistics were used to analyze the characteristics of the patients and the score distributions of the 3 BRS items. In addition, percentage floor and ceiling effects (i.e., the percentages of patients scoring at the lowest and the highest scale levels, respectively)^[16,17]^ of the admission BRS data were examined. We calculated the percentages of patients obtaining the highest and the lowest raw sum admission scores on the upper-extremity items and overall motor items. Floor and ceiling effects exceeding 20% were considered notable.^[17,18]^

#### 2.3.2 Unidimensionality

We examined the unidimensionality and Rasch reliability of the upper-extremity items and the overall motor items. We did not examine the lower extremity item (BRS-L) because at least 2 items were required for Rasch analysis.

Rasch analysis with the partial credit model (PCM) was used for examining data–model fitting. Unidimensionality was examined using all BRS admission data. To examine the unidimensionality of the BRS, we used infit and outfit statistics, and principal component analysis (PCA). First, the infit and outfit statistics were used to examine whether the item responses fit the expectations of the PCM model. The values of infit (weighted) mean square (MNSQ) and outfit (unweighted) MNSQ were used as the fit indicators of the model. The acceptable ranges of both infit and outfit MNSQ values for each item are from 0.6 to 1.4.^[19]^

PCA was further applied to examine the standardized residuals (observed BRS scores minus expected scores). The variance explained by BRS items should be large (>50%), while unexplained variance (the residual) in the first contrast should be small (eigenvalue < 2).^[19]^

We also investigated the level of difficulty of the 3 items and the appropriateness of the response category of each item. The level of difficulty was calculated and expressed as a logit score along with Rasch analysis. The appropriateness of the response category was determined by the step difficulties in each item, which should be in order for the design of the response categories to be satisfactory. Disordering of the step difficulties in an item indicates the need for adjustment of the response category.^[20,21]^ In addition, a person–item map was provided that places the step difficulties of the items (the differences between each response category in each item) and person ability along a continuum. Notable gaps along the step difficulty continuum indicate that additional response categories or items are needed to distinguish patients falling in the gaps.

Moreover, we examined whether patients from different subgroups (age groups or sex) but at the same ability level had equal probabilities of responding positively to the three items.^[22]^ We performed the differential item functioning (DIF) analyses for the 3 BRS items. DIF was verified by examining the invariance of item difficulties^[23]^ across different demographic variables, including age (<40, 40 to 64, ≥ 65 years) and sex (male, female). Item bias existed when the DIF contrast was greater than 0.5^[19]^ and the statistical difference was *P* < 0.016 (0.05/3 age groups) or 0.025 (0.05/2 sex groups) for multiple comparisons using Bonferroni correction.^[25]^

#### 2.3.3 Rasch reliability

We examined the Rasch reliability for BRS admission data. We considered Rasch reliability coefficients higher than 0.7 and 0.9 as reliable for group and individual comparisons respectively.^[26]^

#### 2.3.4 The quantification of the BRS

When the upper-extremity items and overall motor items fitted the assumptions of the Rasch model, the sum scores of the upper-extremity items and overall motor items were transformed to Rasch scores, respectively.

#### 2.3.5 Responsiveness

We examined responsiveness using data from patients who completed both BRS and STREAM evaluations on admission and at discharge. The responsiveness was examined using the Rasch scores of the BRS and the STREAM. The following 4 steps were conducted to examine and compare the responsiveness of the BRS and the STREAM. First, the sum scores of the upper-extremity and overall motor items on admission and at discharge were transformed into BRS Rasch scores using Rasch scores obtained by the quantification of the BRS. Second, the sum scores of the subscales of the upper-extremity (UE) and lower-extremity (LE) of the STREAM were transformed into STREAM Rasch scores, as suggested by the study of Hsieh.^[27]^ The Rasch scores of UE and LE were also summed together to represent the overall motor scores of the STREAM. Third, the responsiveness of the upper-extremity motor function and overall motor function of the BRS and the STREAM were examined using 3 indices: paired *t*-test, ES, and SRM. The paired *t*-test was used to determine the statistical significance of the change in scores. The level of statistical significance was set at α = 0.05. The ES is a measure of change obtained by dividing the mean change in scores between assessments on admission and at discharge by the standard deviation (SD) of the assessment on admission. The SRM is the mean change in scores between two measurements divided by the SD of the changes scores. ES and SRM values of 0.20, 0.50, and 0.80 were considered to show small, moderate, and large responsiveness, respectively.^[28]^

Last, to further examine if significant differences existed in the ESs and the SRMs between the BRS and the STREAM, we performed a bootstrap procedure by drawing 1000 random samples with replacement from the original data for the BRS and the STREAM. Each bootstrap sample was the same size as the original sample recruited in the responsiveness analysis. The 1000 bootstrap samples produced 1000 pairs of differences in the ESs and the SRMs of both measures. After sorting these differences from lowest to highest, we examined whether the value 0 was included in the 26th and 975th observations (i.e., 95% confidence intervals [CIs]). If yes, it was considered to indicate no significant difference in the ESs and the SRMs of the BRS and the STREAM.

The descriptive statistics and responsiveness analysis were analyzed using SPSS 17.0 software (SPSS Inc., Chicago, IL). The Rasch analysis was performed using Winstep 3.64.2 software (winsteps.com, Beaverton, OR).^[24]^ The bootstrap procedure was executed using R 3.1.3 software (R Foundation for Statistical Computing, Vienna, Austria).^[29]^

## 3 Results

### 3.1 Characteristics of the participants

**Figure 1:**
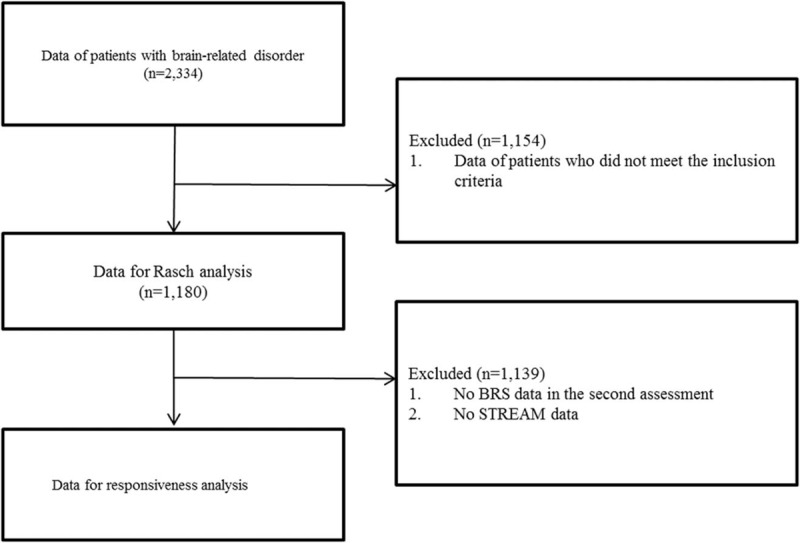
The procedure of data selection in this study.

Figure 1 shows the procedure of the data selection in this study. Admission medical records of 1180 patients with stroke were available. Forty-one subacute patients were further recruited in the responsiveness analysis of the BRS and the STREAM. Table 1 shows the demographic characteristics of the patients. The level distributions of the 3 BRS items are presented in Table 2. Patients who had better motor function (levels V and VI in the 3 BRS items) at admission tended to be lost to follow-up, since patients scoring in levels V and VI in group 1 outnumbered those in group 2. There were no significant differences in patients’ age, sex, diagnosis, or affected sides between patients included and excluded from responsiveness analysis (*P* = 0.061–0.442).

**Table 1:**
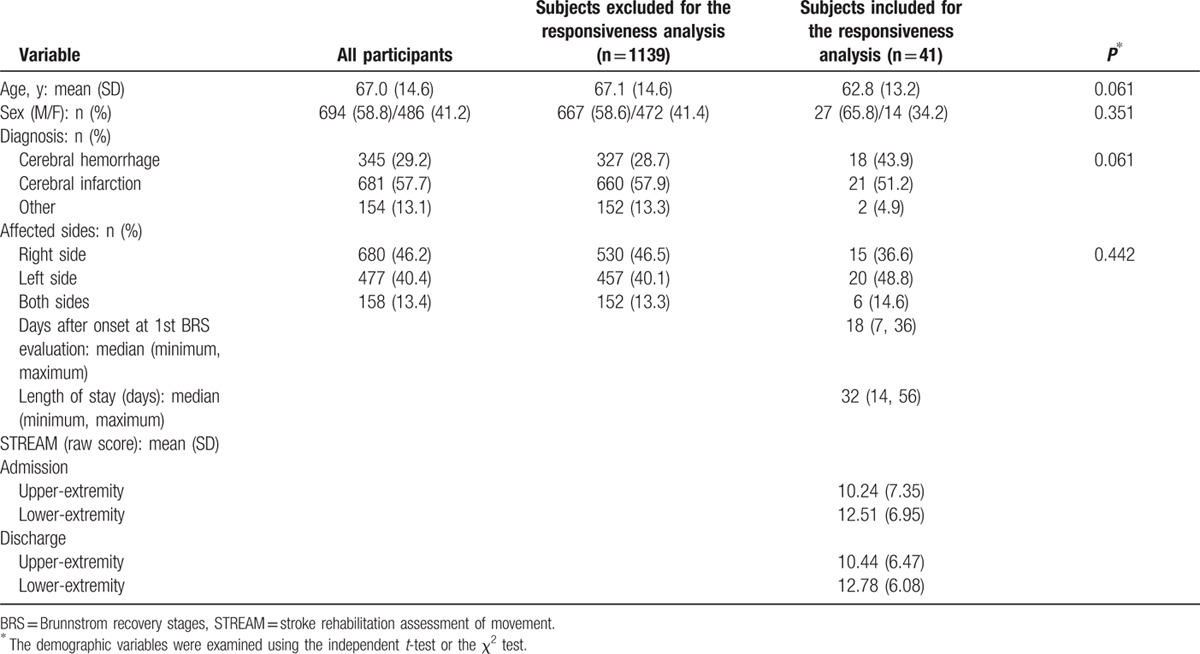
Descriptive characteristics of the patients with stroke.

**Table 2:**
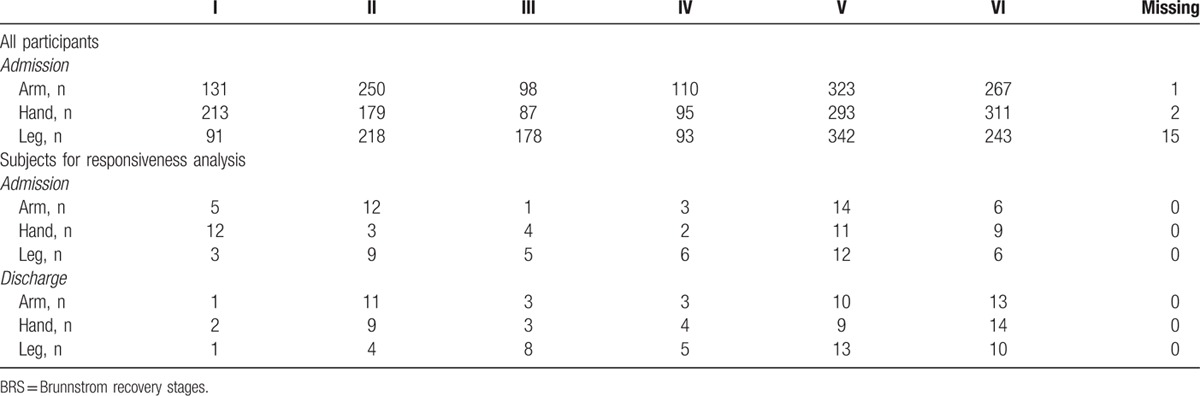
Level distributions of the BRS of participants at admission and discharge (N = 1180).

Only upper-extremity motor function showed a notable ceiling effect, with 20.3% of all patients (n = 239) obtaining the highest scores on admission. There were no obvious floor effects in either upper-extremity motor function or overall motor function.

### 3.2 Unidimensionality

In terms of upper-extremity motor function, both the BRS-A and the BRS-H fitted the model's expectations (infit and outfit MNSQ ranged from 0.80 to 1.06) (Table 3). PCA results showed that 94.7% of the observation variance was explained by both items, and the eigenvalue of unexplained variance in the first contrast was 0.00.

**Table 3:**
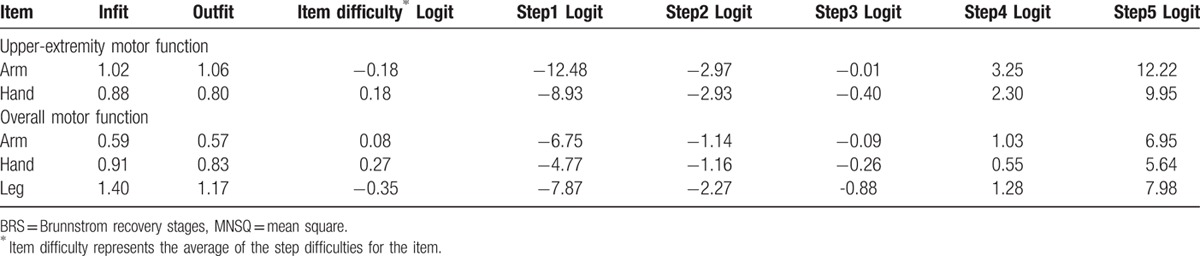
Fit MNSQ statistics, item difficulty, and step parameters of upper-extremity items and overall motor items of the BRS (N = 1180).

Regarding overall motor function, the BRS-H and BRS-L, but not the BRS-A, fitted the model's expectations (infit and outfit MNSQ ranged from 0.83 to 1.40) (Table 3). However, we decided to retain the BRS-A in the BRS because the infit and outfit MNSQ were 0.59 and 0.57, respectively, which were very close to the lower bound of 0.6. PCA showed that 88.0% of the variance was explained by the 3 items, and the eigenvalue of unexplained variance in the first contrast was 1.7.

**Figure 2:**
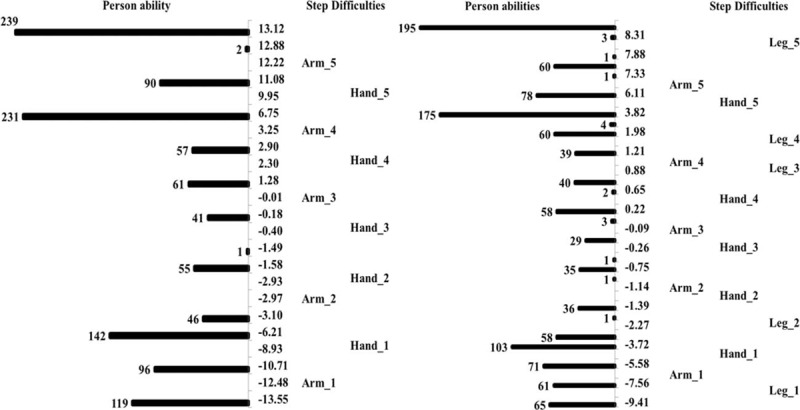
The person–item map of the upper-extremity items (left) and overall items (right) of the BRS. The numbers (such as 1, 2, and 3) next to the items represent the steps of each item; scores in the middle (−13.55 to 13.12 and −9.41 to 9.24) are the logit scores calculated along with Rasch analysis to reflect patients’ motor function or the difficulties of the steps of the items.

The mean difficulties of the 3 items were very close (Table 3). The biggest difference in difficulty among the 3 items was 0.62 logit (BRS-L = −0.35 logit and BRS-H = 0.27 logit). There was no disordering of the step difficulties in the 3 items. The step difficulties among upper-extremity items and overall motor items are shown in the left and right of Figure 2, respectively. For the upper-extremity items, there were 2 notable gaps among steps, one between step 1 of the BRS-H and step 2 of the BRS-A and between step 4 of the BRS-A and step 5 of the BRS-H. Similar results were found for the overall motor items. Examining each step of the 3 items revealed a notable gap between step 5 of the BRS-A and step 5 of the BRS-L.

No significant DIFs by age and sex were found, indicating that the difficulties of each item were the same across patients in all age groups and both sexes (*P* = 0.05–0.29).

Accordingly, the 3 items of the BRS were unidimensional. In addition, the hierarchy of items was identified, which aids in the understanding of progress in the recovery of motor function. No significant DIFs due to age or sex were found.

### 3.3 Rasch reliability

Rasch reliability coefficients of the upper-extremity motor function were 0.91 at admission and at discharge. Rasch reliability coefficients of overall motor function were 0.92 at admission and 0.91 at discharge.

The high Rasch reliability of the BRS indicates that the Rasch scores of the BRS were precise for both individuals and groups with stroke.

### 3.4 The quantification of the BRS

Table 4 shows the raw sum scores of upper-extremity items and overall motor items, the corresponding Rasch-transformed scores, and standard errors. Higher scores indicated better motor function. The Rasch scores were dispersed, ranging from −13.5 to 13.1 for upper-extremity motor function, and from −9.4 to 7.6 for overall motor function, indicating that these patients had very different levels of motor function.

**Table 4:**
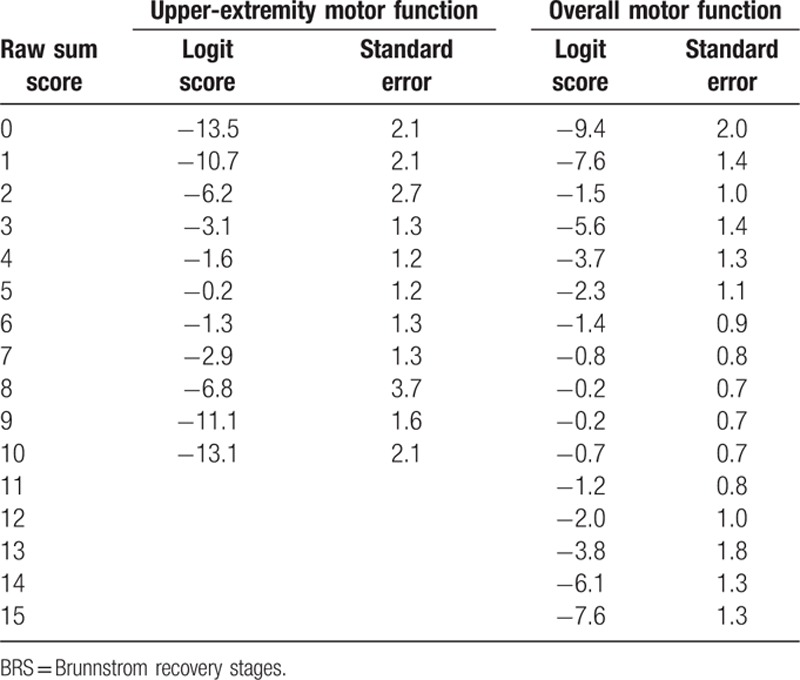
Raw sum score, logit score, and standard error of BRS.

In addition, because the BRS contains only 3 items, the standard errors of Rasch-transformed scores were large, ranging from 1.2 to 3.7 for each score of upper-extremity motor function, and from 0.7 to 2.0 for overall motor function.

Accordingly, the raw sum scores of the BRS have been transformed into Rasch scores to represent the motor function of the upper extremities and overall motor function. Moreover, the standard errors of each Rasch score have also been identified.

### 3.5 Responsiveness

In terms of responsiveness of the BRS, the change scores in both upper-extremity and overall motor functions were significant between admission and discharge (*P* < 0.001). Both upper-extremity motor function and overall motor function had small to large responsiveness (ES = 0.35–0.41, SRM = 0.85–0.99) (Table 5).

**Table 5:**
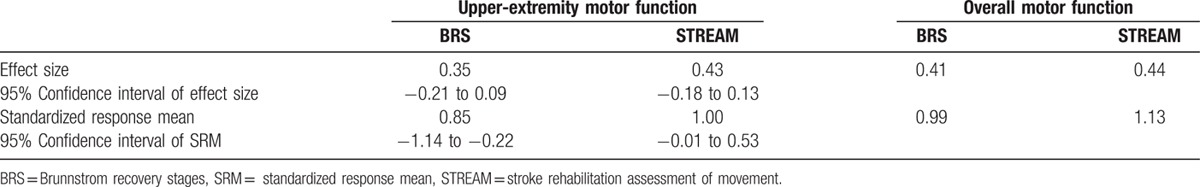
Responsiveness of the BRS and the STREAM.

Regarding the responsiveness of the STREAM, significant differences were also found in the change scores of both upper-extremity and overall motor functions (*P* < 0.001). Both upper-extremity motor function and overall motor function had small to large responsiveness (ES = 0.43–0.44, SRM = 1.00–1.13) (Table 5).

For the comparison of responsiveness between the BRS and the STREAM, the values of ES did not show a significant difference between the BRS and the STREAM (95% CI for difference in ES: from −0.21 to 0.09), whereas the values of SRM showed a significant difference between the BRS and the STREAM (95% CI for difference in SRM: from −1.14 to −0.22) (Table 5).

In summary, the responsiveness of the BRS was acceptable, and generally comparable to the STREAM.

## 4 Discussion

The upper-extremity items (BRS-A and BRS-H) and overall motor items (BRS-A, BRS-H, and BRS-L) both fitted the assumptions of the Rasch model. The results have 2 implications. First, the upper-extremity items and the overall motor items are both unidimensional, supporting the use of their raw sum scores to respectively represent upper-extremity motor function and overall motor function. Therefore, in addition to each item score describing the hand, arm, or leg motor function of a patient, users now have other choices to quantify the patient's poststroke motor function based on their needs (such as presenting an overall motor function or investigating the treatment effect on overall motor function). The second implication is that the raw sum scores of the BRS can further be transformed into Rasch logit scores, as provided in Table 4. As shown in Table 4, all of the adjacent raw sum scores have different logit intervals, calculated by the subtraction of their corresponding Rasch scores. These findings indicate that assessing patients’ progress or comparing the differences of motor function using raw sum scores may result in over- or underestimation of patients’ progress or differences. Therefore, the Rasch scores of the upper-extremity items and overall motor items appear more appropriate for reflecting the extent of upper-extremity motor function and overall motor function, monitoring progress, and comparing the differences of upper-extremity motor function and overall motor function. The Rasch scores of the BRS are strongly recommended for future users.

The DIF analysis revealed no significant differences in item difficulties for patients across different groups (e.g., different age groups and sexes). The results indicated the same performance patterns in the 3 items of the BRS for patients across different subgroups. Thus, the estimated item difficulties are suitable for use with patients across different subgroups.

Rasch analysis provides the Rasch reliability for each patient's score and the overall Rasch reliability (the average of all patients’ reliabilities). The overall Rasch reliabilities of the upper-extremity motor function and overall motor function were above 0.9, with 80% and 77% of the patients having reliability > 0.9 on upper-extremity items and overall motor items, respectively. The Rasch reliability is estimated by the measurement error.^[30]^ Higher reliability represents smaller measurement error, that is, the measure is more precise. A Rasch reliability of a measure higher than 0.7 indicates that the measure is appropriate for groups of patients. That is, the measure is useful for research purposes. In research contexts, the outcomes of patients are often calculated by averaging the measurement scores, which could reduce the effect of measurement errors on the results.^[31]^ A Rasch reliability of a measure higher than 0.9 indicates that the measure is suitable for individual patients. Such a high reliability is especially useful for clinical practice, where measurement of an individual patient is usually followed by a specific decision for that patient.^[31]^ Since the BRS has small measurement error (high Rasch reliability), clinicians can have confidence in using the BRS motor scores to further plan a corresponding intervention protocol and make clinical decisions. Because the BRS has high Rasch reliability, as well as good item-level inter-rater and intra-rater reliability,^[6]^ as supported by previous studies, the BRS can be used to consistently and precisely assess poststroke motor function.

We examined the responsiveness of the BRS using Rasch scores and found small (indexes of ES) or large (indexes of SRM) responsiveness for both upper-extremity and overall motor function. The results indicate that the BRS has sufficient ability to detect the amount of changes in upper-extremity motor function and overall motor function in a group of subacute patients with stroke. Furthermore, we compared the responsiveness of upper-extremity and overall motor function of the BRS to that of the STREAM. No significant differences were found in 3 out of the 4 indexes of responsiveness, indicating that the responsiveness of the BRS is generally comparable to that of the STREAM. These findings are consistent with those of previous studies, which found that for a group of patients, the short-form and long-form measures presented similar responsiveness.^[9,32,33]^ Thus, the BRS has great potential for use as an outcome indictor.

An important finding on the estimated item difficulty and step difficulties should be noted. For the BRS items, no disordering exists in the step difficulties (i.e., higher response categories corresponded to higher level of difficulties). Absence of disordering indicates that the ordinal numbering of categories accords with their fundamental meaning. Therefore, the 6 response categories are appropriate for the BRS. However, obvious gaps exist between the step difficulties of each item. The gaps between the step difficulties indicate a lack of appropriate items or response categories to discriminate a patient's motor functions within the gaps. Therefore, we suggest that additional response categories be used to differentiate upper-extremity motor function and overall motor function in patients with stroke who fall in the gaps of the current scale. For example, an additional response category could be added to each BRS item to differentiate those patients whose motor function falls between levels V and VI.

Compared with the sample size used in Rasch analysis (n = 1180), the sample size used in the responsiveness analysis was relatively small (n = 41). Such a sample size was acceptable but may have restricted the external inference of the responsiveness results. The sample size was acceptable because, with our sample size and the large ES (≥ 0.90), the power of paired *t*-test analysis was 0.99. Moreover, the calculation of ES and SRM is free of the influence of sample size.^[28]^ Therefore, although the sample size looked modest, our results still provided certain evidence of the responsiveness of the BRS. However, these 41 patients were subacute patients (days after onset at 1st BRS evaluation: 7 to 36 days), and the numbers of people in the levels of each item were not evenly distributed (e.g., only 1 person was in level III in the arm item), which might have restricted the representativeness of our sample. More studies are suggested to cross-validate the responsiveness of the BRS in samples with different characteristics, such as acute or chronic patients.

Our study provided sufficient evidence on the psychometric properties of the BRS despite the retrospective design. Our study had 2 strengths, which provide robust evidence supporting the unidimensionality and Rasch reliability of the BRS. First, a large sample size (n = 1180) was used in Rasch analysis, providing stable estimations of item parameters, such as item and step difficulties. Second, our consecutive sampling method increased the representativeness of our sample for patients with stroke. All patients with stroke in the occupational therapy department of a medical center were recruited during the period of 2012 to 2014, except those with comorbidities affecting motor function. Nevertheless, a weakness of our study was the use of a retrospective design, which made missing data inevitable. In addition, multiple raters of the BRS might have increased the measurement error and underestimated the psychometric properties of the BRS. However, our results indicated positive findings. Thus, the aforementioned weaknesses might not be of concern.

This study has 3 contributions. First, we added an extra value to the BRS, that is, transformed the BRS from the ordinal measure into an interval measure. The BRS could preserve the original characteristics such as high accessibility and efficiently identify the motor function of patients’ upper and lower extremities. Moreover, the BRS can accurately reflect changes within a patient, and difference between patients. Second, we again verified that the BRS could be a useful assessment tool of poststroke motor function. For practitioners in areas that seldom use the BRS, practitioners have one more option of assessment tool to assess poststroke motor function. Third, for practitioners in areas that widely use the BRS such as Asia, practitioners could have better interpretations of scores of the BRS.

A limitation of this study should be noted. Upper-extremity motor function contains only 2 items. The low number of items in upper-extremity motor function may have reduced the precision of estimates (greater standard errors) in transformed Rasch scores, as mentioned in the Results section, subsection “The quantification of the BRS.” Therefore, the interpretations of the results of the transformed interval scores of upper-extremity motor function should be conservative.

## 5 Conclusion

This study had 2 aims: first, to examine the psychometric properties of the BRS, and second, to transform the ordinal scale of the BRS into an interval scale to represent a patient's motor function. Our results supported the psychometric properties of the BRS, that is, the unidimensionality, high Rasch reliability, and acceptable responsiveness. Moreover, the ordinal sum scores of the BRS could be transformed into interval Rasch scores. Therefore, prospective users could use Rasch scores of the BRS to represent a patient's overall motor function, and to precisely quantify both changes within a patient and differences between patients.
